# Prevalence and Risk Factors of Anemia in the MASHAD Cohort Study

**DOI:** 10.34172/aim.2023.47

**Published:** 2023-06-01

**Authors:** Maryam Tayefi, Mahmoud Ebrahimi, Sara Saffar Soflaei, Sania Saljoughian, Najmeh Jaberi, Majid Khadem Rezaiyan, Farzad Rahmani, Mohsen Moohebati, Habibollah Esmaily, Seyed Mohammad Reza Parizadeh, Reza Heidari-Bakavoli, Mohammad Safarian, Mohsen Nematy, Mahmoud Reza Azarpazhooh, Gordon A. Ferns A. Ferns, Majid Ghayour-Mobarhan

**Affiliations:** ^1^Biochemistry of Nutrition Research Center, Faculty of Medicine, Mashhad University of Medical Sciences, Mashhad, Iran; ^2^Cardiovascular Research Center, Faculty of Medicine, Mashhad University of Medical Sciences, Mashhad, Iran; ^3^International UNESCO Center for Health-Related Basic Sciences and Human Nutrition, Mashhad University of Medical Sciences, Mashhad, Iran; ^4^Department of Modern Sciences and Technologies, Mashhad University of Medical Sciences, Mashhad, Iran; ^5^Department of Community Medicine and Public Health, School of Medicine, Mashhad University of Medical Sciences, Mashhad, Iran; ^6^Department of Biostatistics and Epidemiology, School of Health, Mashhad University of Medical Sciences, Mashhad, Iran; ^7^Social Determinants of Health Research Center, Mashhad University of Medical Sciences, Mashhad, Iran; ^8^Brighton & Sussex Medical School, Division of Medical Education, Falmer, Brighton, Sussex BN1 9PH, UK; ^9^Metabolic Syndrome Research Center, Faculty of Medicine, Mashhad University of Medical Sciences, Mashhad, Iran

**Keywords:** Anemia, Cardiovascular disease, Diabetes mellitus, Metabolic syndrome, Prevalence

## Abstract

**Background:**

Anemia is a serious public health problem which may be associated with cardiovascular diseases (CVDs) and brain damage. This survey aims to determine the prevalence of anemia and its association with demographic and biochemical factors and metabolic syndrome in a human sample derived from the MASHAD cohort study.

**Methods:**

This survey was conducted on a sub-sample of 9847 individuals aged 35 to 65 as part of the MASHAD cohort study. Demographic characteristics and biochemical and anthropometrics indices were recorded. Data were analyzed using SPSS version 20.

**Results:**

Anemia was seen in 11.5% of the population. Anemia was significantly more prevalent in younger subject (*P*<0.001), females (*P*<0.001) and those with elevated body mass index (BMI) (*P*<0.001). Mean high-density lipoprotein (HDL) was higher in anemic participant (*P*=0.032). The incidence of anemia was significantly lower in smokers (*P*<0.001) and also participant with hypertension (HTN) (*P*<0.001), diabetes mellitus (DM) (*P*<0.001) and metabolic syndrome (MetS) (*P*<0.001). Mean FBG (*P*<0.001), TG (*P*<0.001), total cholesterol (*P*<0.001), LDL (*P*<0.001) and uric acid (*P*<0.001) were significantly lower in anemic subjects. Cholesterol, MetS, low-density lipoprotein (LDL), BMI, uric acid, diabetes mellitus and also TG remained significantly different after multivariate analysis between anemic and healthy participants.

**Conclusion:**

The studied population had a lower prevalence of anemia compared to the previous WHO report for Iranians. Iron deficiency is recognized as the most important cause of anemia in Iran; however, further investigations will be need to confirm this pattern. We demonstrated that anemia is adversely associated with MetS and DM.

## Introduction

 Anemia is defined by a condition in which the count of healthy RBC or oxygen-carrying capacity is not sufficient to meet physiological needs. It is affected by sex, age, height, smoking, chronic diseases and pregnancy status.^1^ Base on the WHO, anemia is defined as hemoglobin (Hb) lower than 120 mg/dL and 130 mg/dL in females and males, respectively. Although iron deficiency is recognized as the most important cause of anemia worldwide, other conditions, such as deficiency in folate, vitamin B12 and vitamin A, chronic diseases and inflammation, parasitic infections, and inherited disorders can also cause anemia.^2^ Thus, anemia is the consequence of impaired nutrition and poor health.^3^

 Although, mean Hb concentrations and the prevalence of anemia are not distributed uniformly in different regions and countries, anemia is more prevalent in women in almost all regions and for all different age groups.^4^ The highest prevalence of low HB levels pertains to East, West and sub-Saharan Africa. Iron-deficiency is the most prevalent cause of anemia in most populations. Kassebaum et al estimated the worldwide prevalence of anemia at 32.9% in 2010, leading to approximately 68.4 million years lived with disability (YLD).^5^ The Global Burden of Disease (GBD) study reported that anemia accounted for 1% of disability-adjusted life-years and 2% of all YLD.^6^ These results highlight the importance of attention to anemia for overall GBD and also show that special consideration should be made in this regard.

 The most alarming health effect of anemia is the increased risk of maternal and child mortality due to severe anemia.^7^ In 2011, 29% (496 million) of non-pregnant women and 38% (32.4 million) of pregnant women aged 15-49 years were anemic.^8^ Anemia is a major health problem mainly affecting developing countries. The WHO reported that the highest prevalence of anemia in all the population groups across the world pertained to the African, South East Asian and Eastern Mediterranean regions (EMR) in 2011. Furthermore, the greatest number of anemic females and children live in the South-East Asia Region; including 202.0 million anemic females in childbearing age and 96.7 million anemic children in 2011. According to WHO reports, 28% of Iranian females were anemic, with mostly mild to moderate anemia. Only 0.8% of reported anemia in Iran was severe (Hb < 80 mg/dL).^2^

 Anemia is often a treatable public health problem, but it may be associated with heart and brain damage if not treated appropriately.^9^ Untreated anemia may also increase the rate of morbidity in other conditions including cardiovascular diseases (CVDs).^10,11^ However, the potential mechanisms linking anemia to increased risk of mortality in CVD has not yet been fully explained, although the NHLBI has reported that reduced oxygen delivery to all organs and tissues is the most important outcome of anemia in CVD.

 Anemia reduction is the world nutrition goal for 2025, which aims for a fifty percent decline in anemia among women in childbearing age.^12^

 In this cross-sectional survey, we aimed to investigate the anemia prevalence among an adult group in a north-eastern region of Iran and investigate the association between anemia and biochemical factors. Also, we studied the relationship between anemia and CVD, hypertension (HTN) and metabolic syndrome (MetS).

## Materials and Methods

 The data for the current study was part of the Mashhad stroke and heart atherosclerotic disorder study (MASHAD); a cohort study which started from 2010 and was designed to investigate various CVD risk factors among adults in Mashhad, Iran who did not have any chronic disease at baseline.^13^ Individuals were selected from the MASHAD study, using stratified cluster random sampling methods. A total of 9847 subjects aged 35 to 65 years were recruited. Using a questionnaire, we recorded baseline characteristics and medical history including smoking status, history of diabetes, HTN and hyperlipidemia. Systolic and diastolic pressures were measured in the upright position. Anthropometric indices including height and body weight were determined at baseline and body mass index (BMI) was calculated with the formula(*weight (kg)/(height (m)*^2^). After 14 hours of fasting, blood samples were taken through the ante-cubital vein. A complete blood count, including Hb, RBC, mean corpuscular hemoglobin concentration (MCHC), mean corpuscular volume (MCV), mean corpuscular hemoglobin (MCH), hematocrit (HCT) and platelets (PLT), was performed by Sysmex K21.The level of carbohydrates (CHO), fasting blood glucose (FBG), triglyceride (TG), high-density lipoprotein (HDL), low-density lipoprotein (LDL) and uric acid were measured with Pars Azmoon kits (mg/dL). Based on Hb, the participants were categorized in two groups: non-anemic (Hb ≥ 12 mg/dL in females and ≥ 13 mg/dL in males) and anemic (Hb < 12 mg/dL in females and < 13 mg/dL in males).

###  Definitions

 HTN was defined as systolic blood pressure (SBP) equal to or greater than 140 mm Hg and/or diastolic blood pressure (DBP) equal to or greater than 90 mm Hg, or taking anti-HTN drugs. Diabetes mellitus (DM) was defined as fasting blood sugar (not drinking or eating anything except water for 8 hours before the test) equal to or greater than 126 mg/dL or drug therapy with insulin or hypoglycemic medications. The components of MetS were defined according to the IDF criteria:^14^ Central obesity (waistline ≥ 80 cm for women or ≥ 94 cm for men) with at least two of these four criteria: 1) HDL lower than 40 mg/dL in males and lower than 50 mg/dL in females or medication therapy for low HDL; 2) TG equal to or greater than 150 mg/dL or medication therapy for elevated triglycerides; 3) FBG equal to or greater than 100 mg/dL or previous diagnosis of type 2 diabetes; 4) SBP equal to or greater than 130 or DBP equal to or greater than 85 mmHg or hypertensive medical therapy.

 The cut-off values for CBC indices were taken from Goldman’s Cecil medicine^15^: {RBC count ( × 106/µL): (low level < 4.5 in males and < 4 in females), (normal level: 4.5-6 in male and 4-5.4 in female), (high level > 6 in males and > 5.4 in females)};{MCV (fL): (low level < 80, normal level: 80-100, high level ( > 100) };{MCH (pg): (low level < 27), (normal level: 27-31), (high level > 31)}; {MCHC(g/dL): (low level < 30), (normal level: 30-36), (high level > 36)}; {HCT(%):(low level < 40 in males and < 36 in females), (normal level: 40-52 in males and 36-48 in females),( high level > 52 in males and > 48 in females)}; {PLT: (low level < 150), (normal level: 150-450), (high level > 450)}.

###  Statistical Analysis

 Statistical analysis was carried out using the IBM SPSS software version 20.0 (IBM cooperation, Chicago, IL, USA). To evaluate normality of data, Kolmogorov-Smirnov test was used. Descriptive analysis was used to process the outcomes in tables and graphs. Data were presented as median and interquartile range (for non-normally distributed data) and mean ± standard deviation (for normally distributed data). ANOVA and independent sample t-test were used for normally distributed quantitative variable. Mann-Whitney test was used for non-normally distributed quantitative variables and chi-square test for qualitative variables. Logistic regression analysis was used to assess the relationship between anemia and biochemical markers, and odds ratios and 95% confidence intervals were calculated. *P* values less than 0.05 were considered as significant.

## Results

 Out of the total 9847 subjects, 8715 (88.5%) were not anemic, while 1132 (11.5%) were anemic. Baseline characteristics of the population are shown in Table 1. Anemia was significantly more prevalent among females (N:880, 77.7%) compared with males (N:252, 22.3%) (*P* < 0.001). The median age of individuals was significantly higher in the non-anemic group compared to those with anemia (*P* < 0.001). BMI was significantly higher in the anemic subjects compared to the non-anemic subjects (*P* < 0.001). Non-smokers had a higher prevalence of anemia than smokers. Diabetes, MetS and HTN were less likely in anemic patients (*P* < 0.001).

**Table 1:** Baseline Characteristics of the Study Population

**Anemia**		**Non-anemic (n=8715)**	**Anemic (n=1132)**	***P***** Value**
Gender^a^	Male (n)	3700 (42.5%)	252 (22.3%)	**<0.001**
	Female (n)	5015 (57.5%)	880 (77.7%)	
Age (year) (Mean ± SD)^b^		48.31 ± 8.32	46.25 ± 7.47	**<0.001**
BMI(Kg/m^2^) (Mean ± SD)^b^		27.85 ± 4.72	28.26 ± 4.98	**0.009**
Smoking^*^	Non	5908 (67.9%) 894 (10.3%) 1903 (21.9%)	831 (73.4%) 82 (7.2%) 219 (19.3%)	**<0.001**
	Ex			
	Current			
Diabetes mellitus (%)^a^	Without	7270 (85.2%)	994 (88.8%)	**0.001**
	With	1261 (14.8%)	125 (11.2%)	
Metabolic syndrome (%)^a^	Without	5284 (60.8%)	739 (65.3%)	**0.003**
	With	3410 (39.2%)	392 (34.7%)	
HTN (%)^a^	Without	5846 (68%)	799 (71.5%)	**0.016**
	With	2756 (32%)	318 (28.5%)	

BMI, body mass index; HTN, hypertension. ^a^Analyzed by crosstabs, ^b^Analyzed by independent sample t-test. *P* values that are less than 0.05 are in bold.

 TG, CHO and LDL levels were significantly lower (*P* < 0.001) in the anemic groups compared to the non-anemic group, whereas HDL was found to be significantly higher (*P* < 0.001) in individuals with anemia compared to non-anemic subjects. Furthermore, FBG and uric acid were significantly higher in the non-anemic group compared to the anemic group (*P* < 0.001). Serum high-sensitivity C-reactive protein (hs-CRP) was not significantly different between the anemic and non-anemic groups (Table 2).

**Table 2:** Mean & Standard Deviation of Anemic & Healthy Population in Mashhad

**Characters **	**Health Status**		***P***** Value**
	**Anemic population=1132**	**Non-anemic population=8715**	
Total cholesterol (mg/dL)^a^	182.92 ± 39.87	192.44 ± 38.91	**<0.001**
TG (mg/dL)^b^	105 (75-150)	122 (86-175)	**<0.001**
LDL (mg/dL)^a^	110.47 ± 35.19	117.47 ± 35.19	**<0.001**
HDL (mg/dL)^a^	43.48 ± 10.13	42.78 ± 9.92	**0.032**
FBG (mg/dL)^a^	88.35 ± 32.57	93.27 ± 40.09	**<0.001**
Hs-CRP^b^	1.66 (0.98-3.97)	1.63 (1.00-3.50)	0.334
Uric acid (mg/dL)^#^	4.29 ± 1.41	4.71 ± 1.39	**<0.001**

HDL, high‐density lipoprotein; LDL, low‐density lipoprotein; TG, triglyceride; Hs-CRP; high-sensitivity C-reactive protein, FBG; fasting blood glucose. ^a^Analyzed by independent sample t-test and, ^b^Analyzed by Mann-Whitney test. *P* values that are less than 0.05 are in bold.

 All blood indices including RBC, MCV, MCH, MCHC, HCT and PLT were significantly reduced in the anemic group compared with non-anemic subjects (*P* < 0.001) (Table 3). MCV was lower than 80 fL in 48.1% of anemic subjects while it was normal in 51.9% of non-anemic subjects.

**Table 3:** Association Between Severity of Anemia and Complete Blood Count Indices According to Low, Normal and High Values

**Hemoglobin Status**		**Non-anemic (8715)**	**Anemic (1132)**	***P***** Value**
RBC^a^	Low	169 (2.0%)	221 (19.5%)	**<0.001**
	Normal	8288 (97.1%)	870 (76.9%)	
	High	81 (0.9%)	40 (3.5%)	
MCV^a^	Low	526 (6.2%)	554 (48.1%)	**<0.001**
	Normal	8005 (93.8%)	587 (51.9%)	
	high	7 (0.1%)	1 (0.1%)	
MCH^a^	Low	1018 (12.0%)	735 (65.7%)	**<0.001**
	Normal	6919 (81.6%)	363 (32.4%)	
	High	542 (6.4%)	21 (1.9%)	
MCHC^a^	Low	29 (0.3%)	241 (21.3%)	**<0.001**
	Normal	8455 (99.0%)	889 (78.6%)	
	High	54 (0.6%)	1 (0.1%)	
HCT^a^	Low	259 (3.0%)	747 (66.0%)	**<0.001**
	Normal	8246 (96.6%)	384 (34.0%)	
	High	33 (0.4%)	0 (0.0%)	
PLT^a^	Low	567 (6.7%)	76 (6.8%)	**<0.001**
	Normal	7886 (93.1%)	1021 (91.1%)	
	High	16 (0.2%)	24 (2.1%)	

RBC, red blood cell; MCV, mean corpuscular volume; HCT, hematocrit; MCH, mean corpuscular hemoglobin; MCHC, mean corpuscular hemoglobin concentration; PLT, platelet. ^a^Analyzed by crosstabs. *P* values that are less than 0.05 are in bold.

 The prevalence of DM and MetS was significantly lower in the anemic group compared with the non-anemic group after multivariate analysis and adjusted for sex and age, while anemic subjects had higher BMI compared with non-anemic subjects. Also, the anemic group had lower levels of cholesterol, LDL, TG, FBG and uric acid compared with the non-anemic group (Table 4).

**Table 4:** Relative Risk of Anemia in Comparison with the Non-anemic Group Associated with Biochemical Markers (Adjusted for Age and Sex)

**Variables**		**Non-anemic Group and** **Anemic Group [OR (95% CI)]**	***P***** Value**
BMI		0.988 (0.977-0.999)	**0.035**
HTN		1.071 (0.917-1.265)	0.356
Diabetes mellitus		0.798 (0.653-0.975)	**0.030**
Metabolic Syndrome		0.786 (0.686-0.900)	**<0.001**
Smoking^a^	Ex-smoker	0.899 (0.705-1.148)	0.310
	Current smoker	0.945 (0.805-1.110)	0.435
Cholesterol		0.993 (0.991-0.995)	**<0.001**
LDL		0.994 (0.992-0.996)	**<0.001**
HDL		0.998 (0.991-1.004)	0.580
TG		0.997 (0.996-0.998)	**<0.001**
Uric acid		0.890 (0.844-0.936)	**<0.001**

BMI, body mass index; HTN, hypertension; HDL, high‐density lipoprotein; LDL, low‐density lipoprotein; TG, triglyceride; CI, confidence interval; OR, Odds ratio. ^*^Analyzed by binary logistic regression, reference group: non-smokers. *P* values that are less than 0.05 are in bold.

 The results of classifying the anemic and non-anemic groups according to the number of MetS components are shown in Table 5. Most of the individuals in the anemic group had at least two components of MetS. RBC, HCT, MCH and MCHC were significantly higher in subjects with no MetS compared to individuals who had at least one component of this syndrome (Table 5).

**Table 5:** Differences in Anemia and Complete Blood Count Parameters Based on the Number of Positive Features of the Metabolic Syndrome

	**Number of Metabolic Syndrome Components**						***P***** Value**^c^
	**zero**	**One**	**Two**	**Three**	**Four**	**Five**	
Anemia^a^ (%)	22.3	25.5	32.2	27.6	21.4	18.2	**<0.001**
Non anemia^a^ (%)	77.7	74.5	67.8	72.4	78.6	81.8	
RBC^b^ ( × 10^6^/µL)	4.90 ± 0.49 0 vs 1 and 2	4.83 ± 0.50 1 vs 4	4.81 ± 0.48 2 vs 4 and 5	4.85 ± 0.46 3 vs 4	4.95 ± 0.47	4.99 ± 0.44	**<0.001**
MCV^b^ (fL)	85.16 ± 5.40 0 vs 3 and 4	85.56 ± 6.03 1 vs 2, 3 and 4	84.78 ± 6.25 2 vs 3	84.07 ± 6.55	84.25 ± 5.83	84.75 ± 4.79	**<0.001**
HCT^b^ (%)	41.71 ± 3.85 0 vs 1, 2 and 3	41.35 ± 3.96 1 vs 2 and 3	40.77 ± 4.04 2 vs 4 and 5	40.84 ± 3.81 3 vs 4 and 5	41.72 ± 3.97	42.18 ± 3.22	**<0.001**
MCH^b^ (pg)	28.50 ± 2.72 0 vs 2 and 3	28.56 ± 2.49 1 vs 2, 3 and 4	28.22 ± 2.77	27.99 ± 2.65	28.20 ± 2.39	28.13 ± 2.02	**<0.001**
MCHC^b^ (g/dL)	33.37 ± 1.48 0 vs 2 and 3	33.22 ± 1.77	33.16 ± 1.69	33.14 ± 1.70	33.35 ± 1.57	32.91 ± 3.06	**<0.001**
PLT^b^ ( × 10^3^µL)	228.73 ± 60.38 0 vs 1 and 5	222.4 ± 55.5 1 vs 2, 3, 4 and 5	232.85 ± 63.18 2 vs 5	231.7 ± 62.99 3 vs 5	234.16 ± 65.06 4 vs 5	252.37 ± 68.86	**<0.001**

RBC, red blood cell; MCV, mean corpuscular volume; HCT, hematocrit; MCH, mean corpuscular hemoglobin; MCHC, mean corpuscular hemoglobin concentration; PLT, platelet. ^a^Analyzed by crosstabs, ^b^Analyzed by ANOVA. ^c^The *P* value between the groups was significant (less than 0.05 and marked in bold).

## Discussion

 The total prevalence of anemia was 11.5% among participants in the population of MASHAD study. Among females, 15% were anemic which is lower than previously reported by the WHO (28%) for all Iranian females, while among males, only 6.4% were anemic.^2^ In a recent survey in northeastern Iran, Eftekharzadeh-Mashhadi et al reported the total prevalence of anemia at 9.7%: 12.7% in females and 6.2% in males. Their population consisted of 1675 subjects aged 1 to 90 years.^16^ In another study in northwestern Iran on 3035 subjects aged between 16–49 years, the prevalence of anemia was reported at 9.7% among males and females.^17^ Furthermore, smoking was negatively associated with anemia; most of the participants with anemia were non-smokers. This is compatible with the fact that smoking, through causing hypoxia, increases RBC production, HCT and Hb.

 Most of the anemic subjects had normal RBC. Changes in HCT were in line with the prevalence of anemia. Categorizing anemia helps us to diagnose the cause of anemia; using parameters in CBC is common and effective.^15^ Based on MCV which shows the size of RBC, anemia is stratified into microcytic (MCV < 80), normocytic (80 ≤ MCV ≤ 100) and macrocytic (MCV > 100) anemia. In our population, almost 50% of anemic subjects were normocytic. Furthermore, based on the MCHC values, which show the Hb concentration in RBC, anemia may be further divided into hypochromic (MCHC < 30), normochromic (30 ≤ MCHC ≤ 36) and hyperchromic (MCHC > 36) categories. Here, MCHC was lower than the normal range in most of the anemic subjects, suggesting hypochromic anemia in our population. Microcytic RBCs together with hypochromic anemia in our population suggest iron insufficiency as the most important cause of anemia in Iran, and this is compatible with the main cause of anemia worldwide.

 Anemic subjects in our study were more likely to have higher BMI. We found that BMI was higher in moderate anemia compared with the non-anemic and those with mild anemia. Likewise, obesity also has been reported to be associated with anemia in adults in some countries,^18-20^ which may be due to an up-regulation of hepcidin expression in obesity, thereby hampering iron absorption.^21^ We also showed that hs-CRP, which is an indicator of chronic inflammation, was higher in anemic patients which is in line with the findings of a cohort study in outpatients adults.^22^

 However, in our study, most of the subjects with any component of MetS were non-anemic. Sun et al and Jehn et al in two different study populations reported that subjects with higher levels of ferritin were at higher risk of MetS and type 2 diabetes.^23,24^ Low ferritin is an indicator of iron deficiency anemia.^1^ Furthermore, a recent study on male blood donors revealed that serum levels of ferritin and iron are adversely associated with insulin sensitivity,^25^ which is a key element of MetS.^26^ Considering iron deficiency as the main cause of anemia in our subjects based on MCV and MCHC, we showed the negative association between anemia and MetS. Also, we showed that different features of MetS were less prevalent among anemic patients. Furthermore, diabetes mellitus was more prevalent among non-anemic subjects which is comparable with previous studies. Evidence indicates that excessive iron storage increases reactive oxygen species (ROS) which may impair the uptake of insulin and cause insulin resistance.^24,25^ Hence, insulin resistance and ferritin might be the link between the presence of anemia and MetS. In contrast, a study in China indicated that anemia is seriously associated with all features of MetS, although the authors did not indicate any association between ferritin and MetS.^27^ This paradox may be due to the high prevalence of MetS in their studied population.

 The strength of the study relies on the fact that the data was obtained from a large cohort study. In addition, this research reveals the recent prevalence of anemia in regionally or nationally representative populations in Mashhad, Iran. Despite its strengths, this study has several limitations. For instance, we did not examine the nutrition status as an important factor for anemia improvement, including consuming iron supplements or iron-fortified foods. Furthermore, the current study only examines the population of Mashhad, but future research should be conducted on a cohort population from several cities in Iran.

## Conclusion

 In conclusion, in a sample of the population from northeastern Iran, 11.5% showed anemia. Females were more prone to anemia (14.9%) while only 6.3% of males were anemic. Anemic patients tended to be younger and without diabetes, HTN and MetS. Approximately 70% of non-anemic subjects had at least one component of MetS while ~30% of anemic subjects had at least one feature of MetS. We also found that the most common anemia in our population was microcytic hypochromic anemia, which indicates that iron deficiency is the major cause of anemia in our population. Further studies will be needed to confirm the cause of anemia in our population.

## References

[1] Gardner W, Kassebaum N (2020). Global, regional, and national prevalence of anemia and its causes in 204 countries and territories, 1990-2019. Curr Dev Nutr.

[2] Vos T, Abajobir AA, Abate KH, Abbafati C, Abbas KM, Abd-Allah F (2017). Global, regional, and national incidence, prevalence, and years lived with disability for 328 diseases and injuries for 195 countries, 1990-2016: a systematic analysis for the Global Burden of Disease Study 2016. Lancet.

[3] Warner MJ, Kamran MT. Iron deficiency anemia. In: StatPearls. Treasure Island, FL: StatPearls Publishing; 2022.

[4] Abdullah N, Ismail N, Abd Jalal N, Mohd Radin F, Othman R, Kamalul Arifin AS (2020). Prevalence of anaemia and associated risk factors amongst The Malaysian Cohort participants. Ann Hematol.

[5] Kassebaum NJ, Jasrasaria R, Naghavi M, Wulf SK, Johns N, Lozano R (2014). A systematic analysis of global anemia burden from 1990 to 2010. Blood.

[6] Mathers C. The Global Burden of Disease: 2004 Update. World Health Organization; 2008. Available from: https://apps.who.int/iris/handle/10665/43942.

[7] Garzon S, Cacciato PM, Certelli C, Salvaggio C, Magliarditi M, Rizzo G (2020). Iron deficiency anemia in pregnancy: novel approaches for an old problem. Oman Med J.

[8] Stevens GA, Finucane MM, De-Regil LM, Paciorek CJ, Flaxman SR, Branca F (2013). Global, regional, and national trends in haemoglobin concentration and prevalence of total and severe anaemia in children and pregnant and non-pregnant women for 1995-2011: a systematic analysis of population-representative data. Lancet Glob Health.

[9] Chaparro CM, Suchdev PS (2019). Anemia epidemiology, pathophysiology, and etiology in low- and middle-income countries. Ann N Y Acad Sci.

[10] Goel H, Hirsch JR, Deswal A, Hassan SA (2021). Anemia in cardiovascular disease: marker of disease severity or disease-modifying therapeutic target?. Curr Atheroscler Rep.

[11] Fazlinezhad A, Hami M, Shakeri MT, Khatibi-Moghaddam H, Dadgarmoghadam M, Khadem-Rezaiyan M (2017). The relationship between serum hemoglobin and creatinine levels and intra-hospital mortality and morbidity in acute myocardial infarction. Int Cardio Res J.

[12] World Health Organization. Global Nutrition Monitoring Framework: Operational Guidance for Tracking Progress in Meeting Targets for 2025. Available from: https://www.who.int/publications/i/item/9789241513609.

[13] Ghayour-Mobarhan M, Moohebati M, Esmaily H, Ebrahimi M, Parizadeh SM, Heidari-Bakavoli AR (2015). Mashhad stroke and heart atherosclerotic disorder (MASHAD) study: design, baseline characteristics and 10-year cardiovascular risk estimation. Int J Public Health.

[14] Alberti KG, Zimmet P, Shaw J (2005). The metabolic syndrome--a new worldwide definition. Lancet.

[15] Cecil RL, Goldman L, Schafer AI. Goldman’s Cecil Medicine, Expert Consult Premium Edition--Enhanced Online Features and Print, Single Volume, 24: Goldman’s Cecil Medicine. Elsevier Health Sciences; 2012.

[16] Eftekharzadeh-Mashhadi I, Hedayati-Moghaddam MR, Fathimoghadam F, Bidkhori HR, Shamsian SK (2015). Anemia as a public health issue in Mashhad, Iran: evidence from the first population-based study. Acta Med Iran.

[17] Kolahi S, Farzin H, Khoshbaten M (2008). Hypochromic microcytic anemia in Northwestern of Tabriz, Iran. Eur J Gen Med.

[18] Ausk KJ, Ioannou GN (2008). Is obesity associated with anemia of chronic disease? A population-based study. Obesity (Silver Spring).

[19] Karl JP, Lieberman HR, Cable SJ, Williams KW, Glickman EL, Young AJ (2009). Poor iron status is not associated with overweight or overfat in non-obese pre-menopausal women. J Am Coll Nutr.

[20] Qin Y, Melse-Boonstra A, Pan X, Yuan B, Dai Y, Zhao J (2013). Anemia in relation to body mass index and waist circumference among Chinese women. Nutr J.

[21] Cheng PP, Jiao XY, Wang XH, Lin JH, Cai YM (2011). Hepcidin expression in anemia of chronic disease and concomitant iron-deficiency anemia. Clin Exp Med.

[22] Chonchol M, Lippi G, Montagnana M, Muggeo M, Targher G (2008). Association of inflammation with anaemia in patients with chronic kidney disease not requiring chronic dialysis. Nephrol Dial Transplant.

[23] Jehn M, Clark JM, Guallar E (2004). Serum ferritin and risk of the metabolic syndrome in US adults. Diabetes Care.

[24] Sun L, Franco OH, Hu FB, Cai L, Yu Z, Li H (2008). Ferritin concentrations, metabolic syndrome, and type 2 diabetes in middle-aged and elderly Chinese. J Clin Endocrinol Metab.

[25] Borai A, Livingstone C, Farzal A, Baljoon D, Al Sofyani A, Bahijri S (2016). Changes in metabolic indices in response to whole blood donation in male subjects with normal glucose tolerance. Clin Biochem.

[26] Kaur J (2014). A comprehensive review on metabolic syndrome. Cardiol Res Pract.

[27] Shi Z, Hu X, Yuan B, Hu G, Pan X, Holmboe-Ottesen G (2008). Coexistence of anaemia and the metabolic syndrome in adults in Jiangsu, China. Asia Pac J Clin Nutr.

